# Thin water films and particle morphology evolution in nanocrystalline MgO

**DOI:** 10.1111/jace.15775

**Published:** 2018-05-30

**Authors:** Daniel Thomele, Amir R. Gheisi, Matthias Niedermaier, Michael S. Elsässer, Johannes Bernardi, Henrik Grönbeck, Oliver Diwald

**Affiliations:** ^1^ Department of Chemistry and Physics of Materials Paris‐Lodron University Salzburg Salzburg Austria; ^2^ Institute of Particle Technology Friedrich‐Alexander Universität Erlangen‐Nürnberg Erlangen Germany; ^3^ University Service Center for Transmission Electron Microscopy Technische Universität Wien Vienna Austria; ^4^ Department of Physics and Competence Centre for Catalysis Chalmers University of Technology Gothenburg Sweden

**Keywords:** coarsening, grain growth, interfaces, magnesium oxide

## Abstract

A key question in the field of ceramics and catalysis is how and to what extent residual water in the reactive environment of a metal oxide particle powder affects particle coarsening and morphology. With X‐ray Diffraction (XRD) and Transmission Electron Microscopy (TEM), we investigated annealing‐induced morphology changes on powders of MgO nanocubes in different gaseous H_2_O environments. The use of such a model system for particle powders enabled us to describe how adsorbed water that originates from short exposure to air determines the evolution of MgO grain size, morphology, and microstructure. While cubic nanoparticles with a predominant abundance of (100) surface planes retain their shape after annealing to T = 1173 K under continuous pumping with a base pressure of water p(H_2_O) = 10^−5^ mbar, higher water partial pressures promote mass transport on the surfaces and across interfaces of such particle systems. This leads to substantial growth and intergrowth of particles and simultaneously favors the formation of step edges and shallow protrusions on terraces. The mass transfer is promoted by thin films of water providing a two‐dimensional solvent for Mg^2+^ ion hydration. In addition, we obtained direct evidence for hydroxylation‐induced stabilization of (110) faces and step edges of the grain surfaces.

## 1 INTRODUCTION

Water adsorption and subsequent surface reactions can be a key factor for the functionalization and performance of oxide nanomaterials. Depending on the water concentration in the surrounding continuous phase, the film thickness of the adsorption layer covering the nanostructures can be in the range between that of a vacuum/solid and of a bulk liquid/solid interface. This transition is associated with a significant increase in the level of complexity with regard to the physico‐chemical description of a materials system.^1^, ^2^, ^3^, ^4^ At a relative humidity typical for ambient conditions, solid surfaces are covered with water molecules up to a thickness of a few nanometers.^5^, ^6^ The properties of related water layers depend on the surface properties of the substrate^6^ and are typically very different from those of macroscopically thick films. In such a form, water does not only contribute to the conversion of oxide surface layers into hydroxides,^7^ it can also act as a two‐dimensional solvent, which drives the alignment of oxide particles^8^, ^9^ and enables the spontaneous structural and microstructural transformation of particle systems under ambient conditions.^10^, ^11^, ^12^

Knowledge about the chemical and physical stability of MgO‐based nanostructures is of key interest for their processing, for example, for the production of ceramics,^13^, ^14^, ^15^, ^16^ and for their use as materials components at elevated temperatures, such as in catalysis.^17^, ^18^, ^19^, ^20^, ^21^, ^22^, ^23^, ^24^ The interaction of MgO particles with pristine, adsorbate free surfaces with gaseous or liquid water has been studied on particle ensembles in different size regimes.^25^, ^26^ Under such conditions, cubic MgO crystallites first develop (110) truncations, which are composed of monoatomic step edges, and in the following sequence of transformation steps, (111) cuts starting from MgO cube corners.^27^, ^28^

Equally important to stability and functional properties of oxide nanostructures^29^ is the effect of water on the evolution of their morphology during synthesis. MgO particles have been successfully synthesized by a variety of solution‐based processes. Depending on the choice of surface‐active agents and solvents, very different particle morphologies have been obtained.^21^, ^30^, ^31^, ^32^ The different structures result from a combination of kinetic and thermodynamic factors during particle growth across the mixed inorganic–organic interfaces. The growth of MgO nanostructures in the gas phase and at high temperatures is exempt from structure directing surfactants. During Mg combustion in air, MgO nanoparticles grow in the vapor adsorption regime where the coagulation and coalescence of particles do not play any significant role.^33^ Typically, monocrystalline MgO particles with cubic habit are obtained by this process.^34^, ^35^ A recent study reports that admixture of H_2_/Ar to the Mg combustion flame gives rise to MgO particles which appear spherical at the mesoscale.^36^ This type of particle shape is a result of the high abundance of terraces and step edges^37^ rather than (100) planes on the nanoscale.^20^, ^36^, ^38^

From the present study, we learned that adsorbed water originating from the storage of oxide nanoparticle powders in air gives rise to significant particle morphology changes at elevated processing temperatures. Under such conditions, protrusions form on (100) terraces in parallel to the formation of step edges and vicinal surfaces^37^ which, at lower magnifications, give rise to the appearance of rounded grains which are made up from cubic building blocks. Related local structures can accommodate important new functionalities which are beneficial for sintering and microstructure evolution, on the one hand, or related to catalytic activity, on the other.^39^ The here investigated influence of residual surface water on grain morphology may also point to simple, effective and low cost processing routes which represent an important prerequisite for the translation of oxide nanoparticles in functional ceramics and devices.

## 2 METHODS

### 2.1 Experimental methods

MgO nanoparticles were produced in a chemical vapor synthesis reactor at reduced pressure. Details of particle synthesis and subsequent thermal preprocessing are given in References.^40^, ^41^, ^42^ Prior to the different types and annealing procedures, the samples were exposed for twenty minutes to air to allow for adsorption of water molecules from the ambient. Typically 200 mg of MgO nanoparticles are subsequently transferred into a quartz cell which is connected to a high vacuum pumping rack. In the next step the samples were evacuated down to a base pressure of *P* < 10^−5^ mbar and at room temperature for gas removal and for desorption of physisorbed surface species. During sample activation and for the Temperature Programmed Desorption (TPD) experiment (Figure S1), the sample temperature was measured in between the ceramic furnace tube and the quartz cell, while the MgO nanoparticle powder was kept inside the cell at a base pressure < 10^−6^ mbar. The different annealing procedures are run by a computer program based on a PID‐control of the tube furnace temperature (Eurotherm).

Thermogravimetric analysis (TGA) was performed using a Netzsch STA 449 C Jupiter with 10 K min^−1^ heating rate under Argon flow of 20 mL min^−1^ in the temperature range from room temperature (RT) up to 1300 K (sample weight 5 ‐ 7 mg). The samples were transferred from a high vacuum cell immediately or after a certain time of air exposure (p_H2O_ = 7 mbar) to the ceramic crucible (Al_2_O_3_) of the instrument.

For all annealing procedures we used a heating rate of r = 5 K min^−1^ and completed annealing at T = 1173 K at which we switched to annealing in oxygen atmosphere for a period of one hour. We applied three types of annealing procedures which in the following will be designated as (i) dynamic vacuum annealing (DVA), (ii) semi‐dynamic vacuum annealing (semi‐DVA), and (iii) annealing at constant oxygen pressure (Figure S2).

The difference between the procedures is given by the temperatures at which vacuum conditions were switched from dynamic vacuum to a static O_2_ atmosphere of 10 mbar^43^: for dynamic vacuum annealing (DVA) the sample was continuously pumped until T = 1173 K was reached as final temperature (Figure S2). In case of annealing, a constant O_2_ pressure of 10 mbar was kept throughout the entire annealing cycle (Figure S2). In the semi‐dynamic vacuum annealing (semi‐DVA) cycle, we switched at T = 403 K (Figure S2) from dynamic vacuum conditions (continuous pumping at *P* < 10^−5^ mbar) to static gas environment (10 mbar O_2_).

### 2.2 Characterization of structure and morphology

After annealing, the average crystallite size, particle size distribution and morphology were determined using X‐ray diffraction (XRD) and Transmission Electron Microscopy (TEM), respectively. XRD measurements were performed on a Bruker AXS D8 Advance diffractometer using Cu Kα radiation (λ = 154 pm). For the determination of the average domain sizes we applied the Scherer equation to the values of the integral widths of the most prominent (002) and (022) reflexes in the diffraction pattern. For TEM measurements, samples were analyzed by dipping a lacey carbon grid into the powder and using a TECNAI F20 field emission TEM. The micrographs were recorded using a Gatan Orius CCD camera.

### 2.3 Computational method

Density Functional Theory (DFT) was used with the exchange correlation (xc) functional according to Perdew, Burke and Ernzerhof (PBE).^44^ The one‐electron Kohn‐Sham orbitals were expanded in a local numerical basis set. The all‐electron basis functions were centered on the atoms and stored on a radial grid.^45^ (We have used DMol, version 4.0.) The Kohn‐Sham equations were solved with an integration technique based on weighted overlapping spheres centered at each atom. Structural optimization was performed until a convergence criterion of 0.002 eV/Å was met for the largest element of the gradient. Integration over the Brillouin zone was approximated by finite sampling. The lattice constant for MgO was calculated to be 4.26 Å which is a slight overestimation (~2%) with respect to the experimental value.

The effect of water adsorption on the shape of MgO particles was investigated by construction of equilibrium Wulff‐particles using ab initio thermodynamics.^46^, ^47^ Calculations for Mg(110) was done with a *p*(1 × 1) surface cell. The hydroxylated MgO(100) surface has been measured^48^ to have a (3 × 2) superstructure with a mixed H_2_O‐OH monolayer and this structure was considered for MgO(100). As the pristine MgO(111) facet is polar, this was modeled in the (2 × 2) O‐terminated octopole structure,^49^ preventing the polarization catastrophe.^50^

## 3 RESULTS AND DISCUSSION

**Figure 1:**
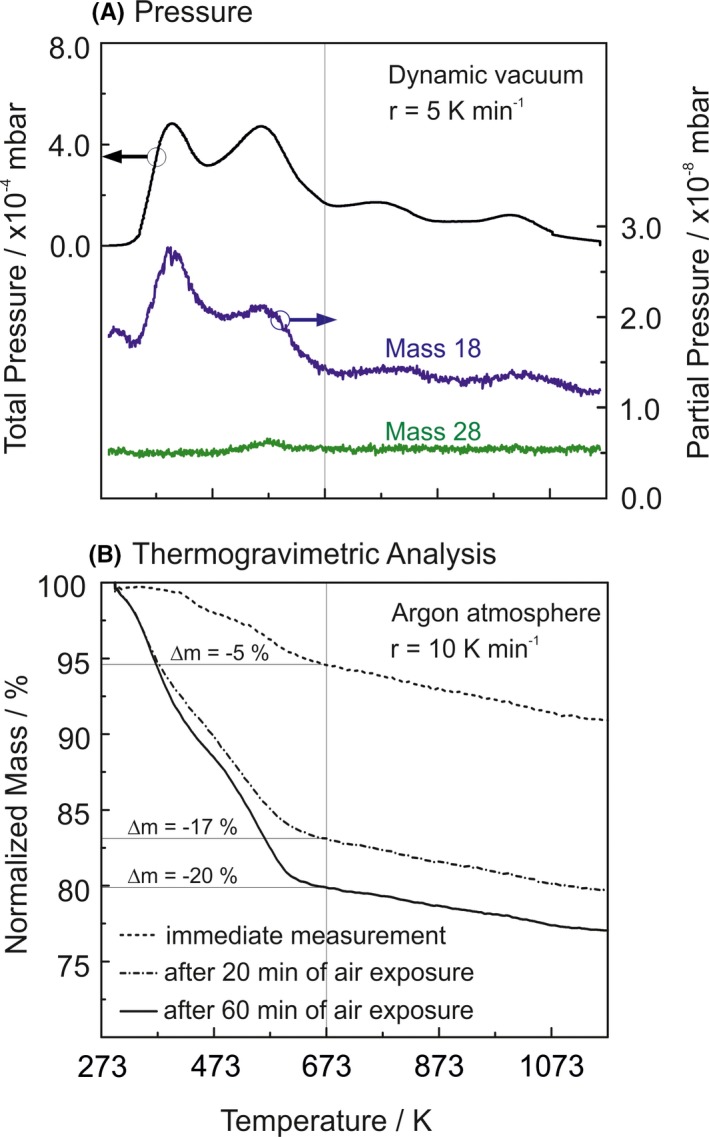
Annealing induced desorption of surface species: (A) black line represents the total pressure increase recorded in the vacuum chamber (left ordinate scale), blue and green lines correspond to the ion currents for mass 18 and mass 28 as determined with the QMS system (right ordinate scale). (B) Thermogravimetric Analysis (TGA) results on MgO nanocube powders after different periods of air exposure [Color figure can be viewed at http://wileyonlinelibrary.com]

Vapor phase grown high surface area materials such as MgO nanocube powders instantaneously adsorb H_2_O from the ambient upon exposure to air. The systematic investigation of the impact of related adsorbates on particle sintering and particle coarsening requires knowledge about nature and amount of the surface adsorbed species, which potentially become surfactants during annealing. Temperature‐Programmed‐Desorption (TPD, Figure 1A) and Thermogravimetric Analysis (TGA, Figure 1B) reveal that depending on the exposure time MgO nanocube powders can adsorb water between 10 and 20 percent of weight.

We performed TPD measurements on MgO nanoparticle powders which ‐ prior to the structural characterization ‐ were exposed to air for 20 minutes at room temperature. A QMS mass spectrometer system (Figure S1) was used for the molecule specific detection of desorbing surface species in the temperature range 273 K ≤ T ≤ 1173 K (Figure 1B).

While the black line in Figure 1A shows the development of the integral pressure with temperature during continuous pumping, the blue curve shows the mass spectrometer signal related to mass 18 amu corresponding to the concentration of water desorbing into the gas phase as a result of nanoparticle powder annealing. The temperature‐dependent pressure changes within the sample cell (Figure 1A) is fully consistent with the changes of water partial pressure in the system (blue curve). Control of the mass range up to 80 amu did not reveal any other significant mass fragments. This suggests that surface dehydration and dehydroxylation and, consequently, H_2_O desorption is the predominant chemical surface process during annealing. The two H_2_O desorption features at T = 303 K and 573 K are attributed to desorption of weakly bound surface water at T = 303 K and to the decomposition of Mg(OH)_2_ into MgO and gas phase water at T = 573 K, respectively. (Although there is a lack of XRD evidence for the presence of crystalline hydroxide phases under these conditions, a minor fraction of surface oxide becomes presumably transformed into amorphous surface hydroxide.)^51^ Two desorption features of lower intensity at T = 773 K and 1023 K are attributed to the elimination of chemisorbed surface hydroxyls. From the TPD profiles (Figure 1) and the TGA results (Figure 1B) we conclude that there is significant water adsorption during room temperature sample exposure to air. An estimate for the number of water molecules per Mg^2+^–O^2−^ surface unit as a function of MgO nanocube size reveals that the number water layers are in the range between 2 and 7 for MgO nanocubes in the size range between 5 and 20 nm (Figure S3).

A large fraction of surface water can be removed by vacuum annealing up to T = 473 K. Moreover, there is no appreciable water desorption above T = 1073 K, a temperature at which surface hydroxyls of highest stability can be eliminated by vacuum annealing.^52^

**Figure 2:**
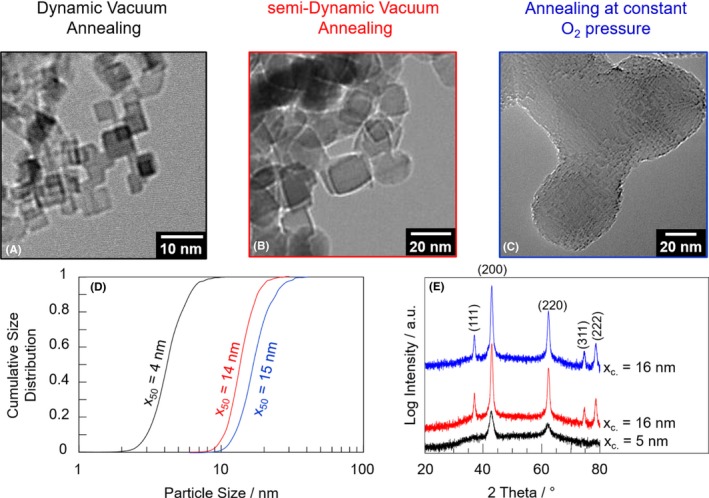
Transmission electron micrographs, particle size distributions and XRD pattern of MgO nanoparticles after annealing according to different annealing protocols or procedures: dynamic vacuum annealing (DVA, (A) black frame and curves), semi‐dynamic vacuum annealing (semi‐DVA, (B) red frame and curves) and static vacuum annealing (anneal at constant oxygen pressure (C) blue frame and curves). The changes in grain size and morphology as concluded from TEM micrographs in 2 (A‐C) are consistent with the respective trends in the size distribution functions (D) and average crystallite domain size (E) as determined from the width of the reflection features using the Scherrer equation [Color figure can be viewed at http://wileyonlinelibrary.com]

The structural analysis of the different particle systems with XRD and TEM revealed substantial differences in terms of average crystallite domain size (XRD), particle size distribution and particle morphology (TEM, Figure 2): DVA powder particles exhibit cubic shape. The ensemble of single crystalline particles is characterized by a narrow size distribution with a modal value of x_50_ = 4 nm which is in good agreement with crystallite domain size of x_c_ = 5 nm. Particles in samples that were subjected to the semi‐DVA protocol grow in size upon annealing, exhibit a median value of x_50_ = 14 nm and a crystallite domain size of x_c_ = 16 nm. MgO nanoparticle powders which were annealed at constant oxygen pressure (i.e., at 10 mbar O_2_ 43) undergo substantial morphology changes (Figure 2). The particles with surface regions that are strongly faceted are rounded. Furthermore, particle fusion and sintering is observed in the TEM micrographs (Figure 2C).

In soft agglomerates of essentially isolated and monocrystalline nanocubes, vacuum annealed MgO nanoparticles (Figure 2A) expose predominantly (100) surface planes to the gas phase. As such they have been extensively described with regard to their surface and interface chemistry in previous publications.^42^, ^53^, ^54^ Here we show that they can be used as reference system for the study of adsorbate mediated coarsening effects, such as those observed for the semi‐DVA particle powders and those annealed at constant oxygen pressure (see below).

**Figure 3:**
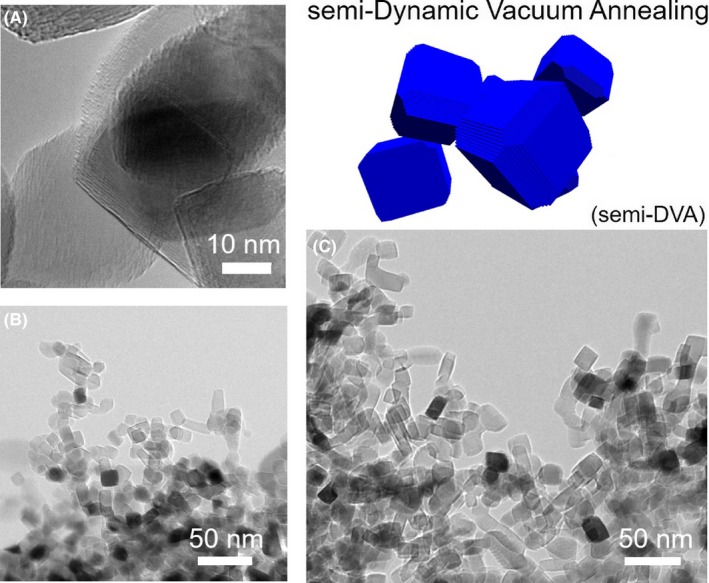
Transmission Electron Micrographs of MgO nanoparticles after semi‐dynamic vacuum annealing (semi‐DVA). The essentially cubic particles exhibit specific surface regions with rounded features. The High Resolution TEM image (A) reveals the actual origin of this effect, that is, a high abundance of step edges and terraces on top of the (100) faces. This leads to rounding effects with regard to the overall particle morphology (B,C) as illustrated by the schematic drawing in the top right panel [Color figure can be viewed at http://wileyonlinelibrary.com]

Application of a semi‐DVA protocol leads to nanoparticle powders that are made up from substantially larger particles (Figure 2D, 3) having larger average crystallite domain size, as determined with the Scherrer equation (Figure 2E). Enhanced particle coarsening must result from facilitated mass transfer through the bulk and/or across the surface of the particles. From the QMS results in Figure 1A, we learned that for the semi‐DVA samples, where we switched at T = 403 K from dynamic vacuum annealing to annealing at a constant oxygen pressure, there remains a substantial amount of water, which is chemically bound to the metal oxide particle surface. As a result and in comparison to DVA particle powder samples, there are also slight but significant changes in the particle shape. The final shape of the semi‐DVA particles can be approximated by a slightly rounded cubic body. A TEM analysis reveals that the underlying particle size and shape is uniform throughout all the samples (Figure 3). This rules out that the shape of the particles results from an oriented attachment process of the different cubes which would lead to staggered structures and multiple edges at regions of original particle contact and coalescence.^8^ Such features and morphological characteristics have not been observed in the course of the TEM investigations of this study (Figure 3).

**Figure 4:**
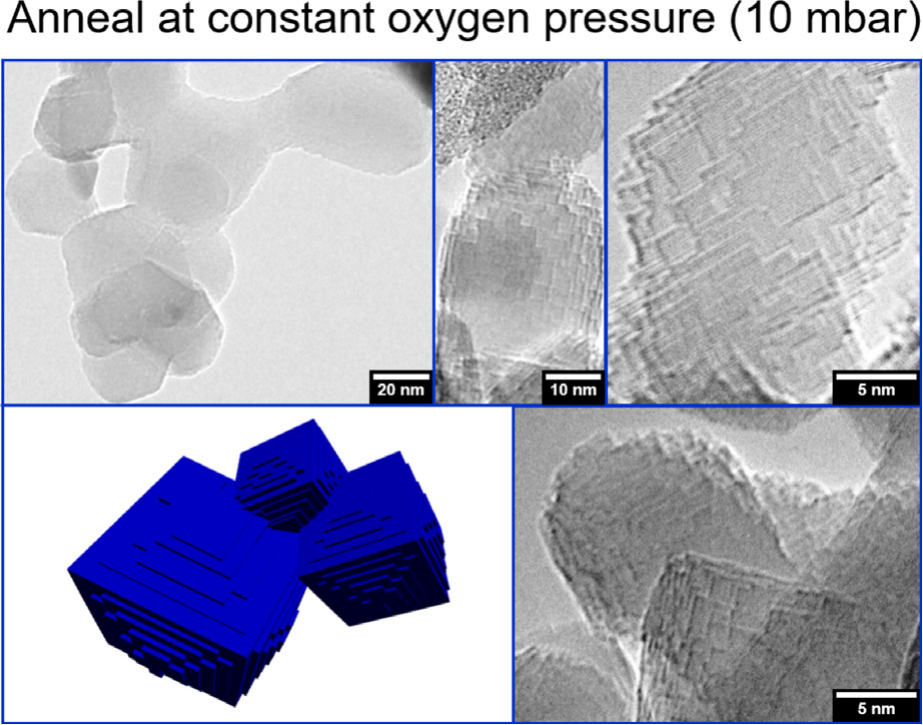
Characteristic TEM and HR‐TEM images of vapor phase grown nanocubes after annealing at constant oxygen pressure (Supplementary Information, Figure S1C). The schematic drawing on the bottom panel on the left side emphasizes the effect of intergrowth and the enhanced abundance of smaller sized terraces and protrusions [Color figure can be viewed at http://wileyonlinelibrary.com]

A detailed inspection of HR TEM images acquired from the semi‐DVA samples shows that the true shape of the somewhat rounded particle body results from hierarchically organized (100) faces of smaller size on top of the MgO(100) cube (Figure 4A).

In addition to an enhanced mass transfer, annealing at constant oxygen pressure (Figure S2) facilitates particle‐particle attachment and neck formation. Ultimately, these processes lead to intergrowth of originally individual and independent coarsened particles. The aggregated and coalesced particles do not exhibit any type of a specific crystallographic orientation relationship with respect to each other. Also at the mesoscale, the samples that were annealed at constant oxygen pressure (10 mbar) are differently organized with respect to the vacuum annealed particle systems. Substantially coarsened grains with a high abundance of step edges, (110) facets, shallow protrusions and terraces were found to form intergrown structures that originate from the aggregation of particles (Figure 4). These changes result from particle aggregation and are further promoted by an enhanced ion transport across the particle‐particle interfaces.

The TPD curve in Figure 1A allows one to discriminate between physisorbed water (with a desorption maximum at 303 K) and water molecules which dissociate upon adsorption and transform the surface oxide into surface hydroxides. The reverse process is characterized by a desorption feature with a maximum at 573 K (Figure 1A). At elevated temperatures, this leads to the release of water which becomes effective during both the semi‐DVA process and during anneal at constant oxygen pressure. The presence of physisorbed water strongly promotes mass transfer and particle intergrowth, a phenomenon that is only observed for the samples subjected to annealing at constant oxygen pressure.

### 3.1 Surface energies and Wulff‐constructions

**Figure 5:**
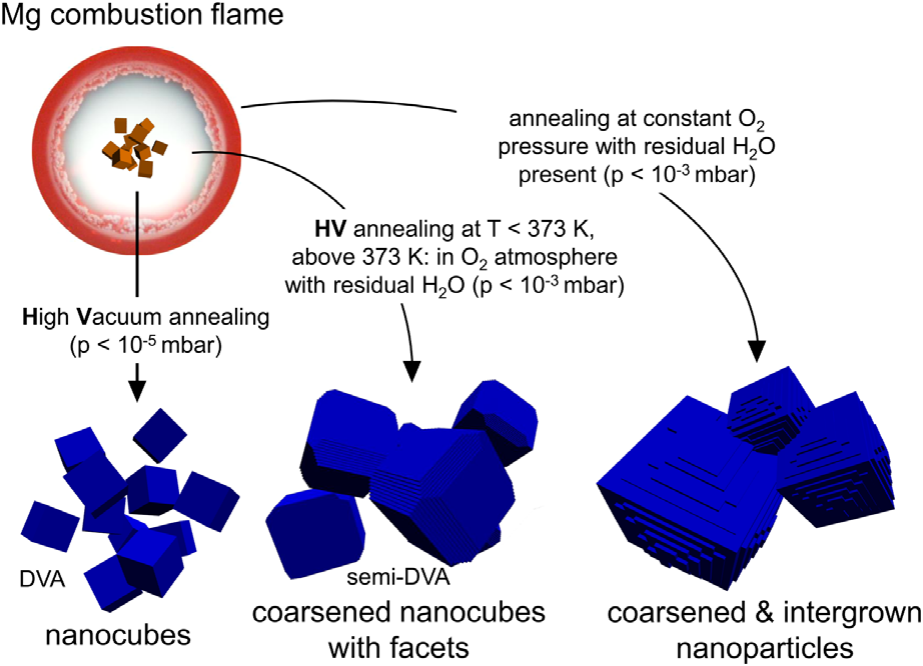
Schematic representation of how different annealing protocols affect the growth of MgO nanocubes synthesized in the Mg combustion flame [Color figure can be viewed at http://wileyonlinelibrary.com]

The different stages of morphology evolution are summarized in Figure 5. For rationalizing the trends observed one needs to consider the impact of thermodynamics explaining the driving forces for the materials transformation together with kinetics and transport limitations. Both types of contributions are strongly interrelated.

**Figure 6:**
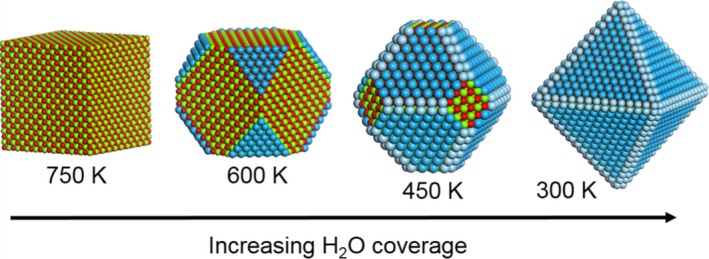
Analysis of the thermodynamic stability of MgO particles at a partial water pressure of 100 Pa as predicted by DFT calculations.^47^ The structural models shows a ~ 4 nm particle. Water molecules are not displayed for clarity and hydroxylated surfaces are instead colored blue. Color code: Mg (green), Oxygen (red), hydroxylated (111) (dark blue), and hydroxylated (110) (light blue) [Color figure can be viewed at http://wileyonlinelibrary.com]

The fact that MgO particles adopt cubic shape during annealing in water‐poor environment, which is experimentally approximated by continuous pumping and a maintained base pressure below 10^−5^ mbar, is a consequence of the clear separation between the surface energies for the low index surfaces of alkaline‐earth metal oxides, see for example,^47^ The surface energy for MgO(100) has been calculated to be 0.93 J/m^2^, whereas the corresponding energies for MgO(110) and MgO(111) were reported to be 2.25 and 2.21 J/m^2^, respectively.^47^ The presence of water, however, may change the relative ordering of the surfaces as a consequence of the stronger adsorption energy to the high index surfaces. The average adsorption energy of H_2_O on MgO(100) and MgO(111) has been reported to be ‐0.73 eV and −2.46 eV, respectively.^47^ Figure 6 shows for a ~ 4 nm particle the thermodynamically stable shapes, as predicted from a Wulff‐construction and at a partial water pressure of 100 Pa. As a result of this modeling experiment the water coverage at high temperatures is low which results in regular cubes as the stable particle shapes. At a constant partial water pressure, lowering the temperature promotes water adsorption yielding faceting and stabilization of water covered MgO(111) and MgO(110).

Although the experimental conditions—there is a temperature induced change in the H_2_O partial pressure (Figure 1A)—are different from those of the theoretical analysis (Figure 6), related observations are consistent with each other. Water promotes faceting and step‐edge formation (Figures 4 and 5) and this trend is enforced with the concentration of adsorbed water which is in good agreement with the thermodynamically determined evolution of particle shape. It should be noted that the presence of the thermodynamically most stable particles could be hindered by high kinetic barriers for the required mass transfer and it is likely that low temperature water adsorption results in situations where also MgO(100) is water covered.

### 3.2 Magnesium dissolution, hydration, and mass transport

The experiments show a pronounced particle coarsening in the presence of water. This effect is attributed to the formation of Mg(OH)_2_ which is followed by dissolution and transportation OH‐solvated Mg^2+^ ions. Ab initio molecular dynamics simulations of multilayer water adsorption on BaO(100) have revealed facile cation dissolution and formation of hydroxylated structures.^55^ Similar phenomena were recently studied for MgO(100), where a Mg(OH)_2_‐layered structure was predicted to be thermodynamically preferred at high chemical potentials of water.^56^, ^57^ Although the barriers for Mg(OH)_2_ formation or detachment of solvated Mg^2+^ from the surface are unknown, it can be assumed that these processes are facile at room temperature. Early measurements, in fact, have suggested that the rate determining step is the detachment of Mg^2+^ ions and an apparent activation energy was measured to be 0.6 eV.^58^

The particle morphology is important for the activity of MgO‐based catalysts.^21^, ^22^, ^24^, ^59^ In the course of the oxidative coupling of methane (OCM) reaction, as an example, where step edges have been suggested to be the active sites.^18^, ^19^, ^60^, ^61^ MgO particle systems were found to undergo sintering as tracked by domain size increase (XRD) and rounding of the previously cubic primary particles (HR‐TEM).^18^ Sharp edges and corners which lend the particles their characteristic cubic shape typically disappear during time‐on‐stream of the chemical reaction upon emergence of {110} and {111} micro facets which are composed of monoatomic and multiple step edges.^18^, ^19^, ^61^

Another interesting aspect relates to the growth of thin film heterostructures, which includes polar oxide/polar semiconductor interfaces and offers exciting opportunities for electronic structure engineering.^62^, ^63^, ^64^ The presence of water vapor during film growth was found to favor layer‐by layer formation and, therefore, promotes the evolution of smooth oxide interfaces and films. As a result of water adsorption, polar {111} metal oxide surfaces become stabilized over a wider range of temperatures and water as a surfactant can provide enhanced control to engineer thin metal oxide based heterostructures.

In this study we have shown the morphological transformations that the MgO nanocubes can undergo during annealing are the result of instantaneous water adsorption that occurs upon nanoparticle exposure to the ambient. Corresponding surface layers of water form thin films, which play an important role for the stability and chemical activity of the oxide nanostructures, which all are soluble to a certain extent. With regard to sintering of nanocrystalline powders and the development of robust and reproducible procedures, one has to take into account that high surface area materials can adsorb a substantial amount of water from the ambient. This surface water may serve as a solvent and sintering agent at elevated temperatures. Moreover, for the molecular interpretation of sintering processes the role of related water films with respect to particle sintering and microstructure evolution requires better understanding.

## 4 CONCLUSIONS

Understanding of morphological transformations at the atomic scale is important because local structures generally determine the functionality of oxide particles as catalysts and other functional materials components. Starting with MgO nanocubes, as particulate model system for surface and interface studies, we have experimentally investigated their morphology evolution in different gaseous H_2_O containing environments. The presence of water strongly affects the MgO grain size, morphology, and microstructure. Cubic nanoparticles with a high abundance of (100) facets are observed after annealing to 1173 K at low water pressures, whereas higher partial pressures of water promote mass transport which leads to particle coarsening, the formation of step edges and shallow protrusions on terraces. The enhanced mass transfer is attributed to the presence of thin films of water, which provide a two‐dimensional solvent for Mg^2+^ ion hydration and transport. The emergence of the above‐mentioned surface structures, on the other hand, are thermodynamically favored at elevated water partial pressures. Our results and related insights are transferable to other metal oxides that exhibit characteristic morphologies at the nanoscale. Moreover, the observation that the moderate exposure of high surface area oxides to water has a substantial impact on grain morphology evolution during sintering may point to simple, effective and low cost processing routes being an important prerequisite for the translation of oxide nanoparticles in functional devices.
