# Supplementary material for: Thin water films and particle morphology evolution in nanocrystalline MgO
Source: J Am Ceram Soc. 2018 May 30;101(11):4994–5003. doi: 10.1111/jace.15775 (PMC6175089; doi:10.1111/jace.15775)
Supplement: Supplementary file 1 [file JACE-101-4994-s001.docx]

Supplementary Information

Thin Water Films and Particle Morphology Evolution

of annealed MgO Nanocrystals

Daniel Thomele^1^, Amir R. Gheisi^2^, Matthias Niedermaier^1^,Michael S. Elsässer^1^,

Johannes Bernardi^3^, Henrik Grönbeck^4^, and Oliver Diwald^1*^

^1^ Department of Chemistry and Physics of Materials, Paris-Lodron University Salzburg, Jakob-Haringer-Strasse 2a, A-5020, Salzburg, Austria;

Email: oliver.diwald@sbg.ac.at

^2^ Institute of Particle Technology, Friedrich-Alexander Universität Erlangen-Nürnberg, Cauerstrasse 4, D-91058, Erlangen, Germany;

^3^ University Service Center for Transmission Electron Microscopy, Technische Universität Wien, Wiedner Hauptstrasse 8-10, A-1040, Vienna, Austria;

^4^ Department of Physics and Competence Centre for Catalysis, Chalmers University of Technology, 412 96 Gothenburg, Sweden

**Table of contents**

1. Set-up for temperature programmed desorption 2
2. Temperature and pressure profiles for thermal annealing 3
3. Estimated film thickness as a function of particle size 4
4. **Set-up for temperature programmed desorption**


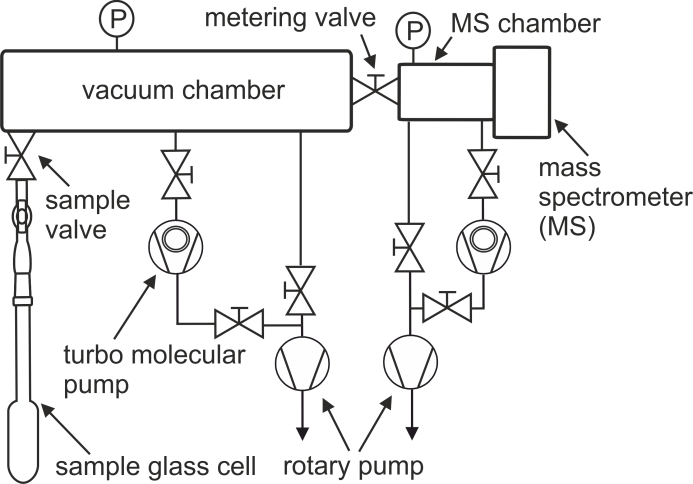


**Figure S1**: Experimental set-up for the detection of annealing induced desorption of species from the surfaces of MgO nanoparticle powders. (P corresponds to pump.)

Temperature and pressure profiles for three types of annealing protocols
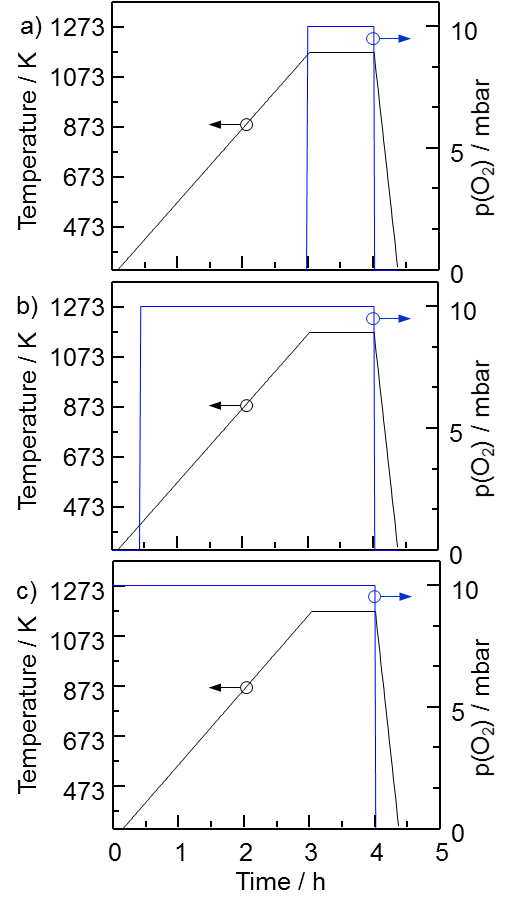


**Figure S2**: Temperature and pressure profile of the three annealing protocols, which were applied to MgO nanocube powders: a) dynamic vacuum annealing (DVA), b) semi-dynamic vacuum annealing (semi-DVA) and c) annealing at constant oxygen pressure (10 mbar). Prior to all annealing steps, the powders were pumped down to a base pressure of p < 10^-5^ mbar at room temperature. The heating rate was kept constant at r = 5 K∙min^-1^.

1. **Estimated film thickness as a function of particle size**

**
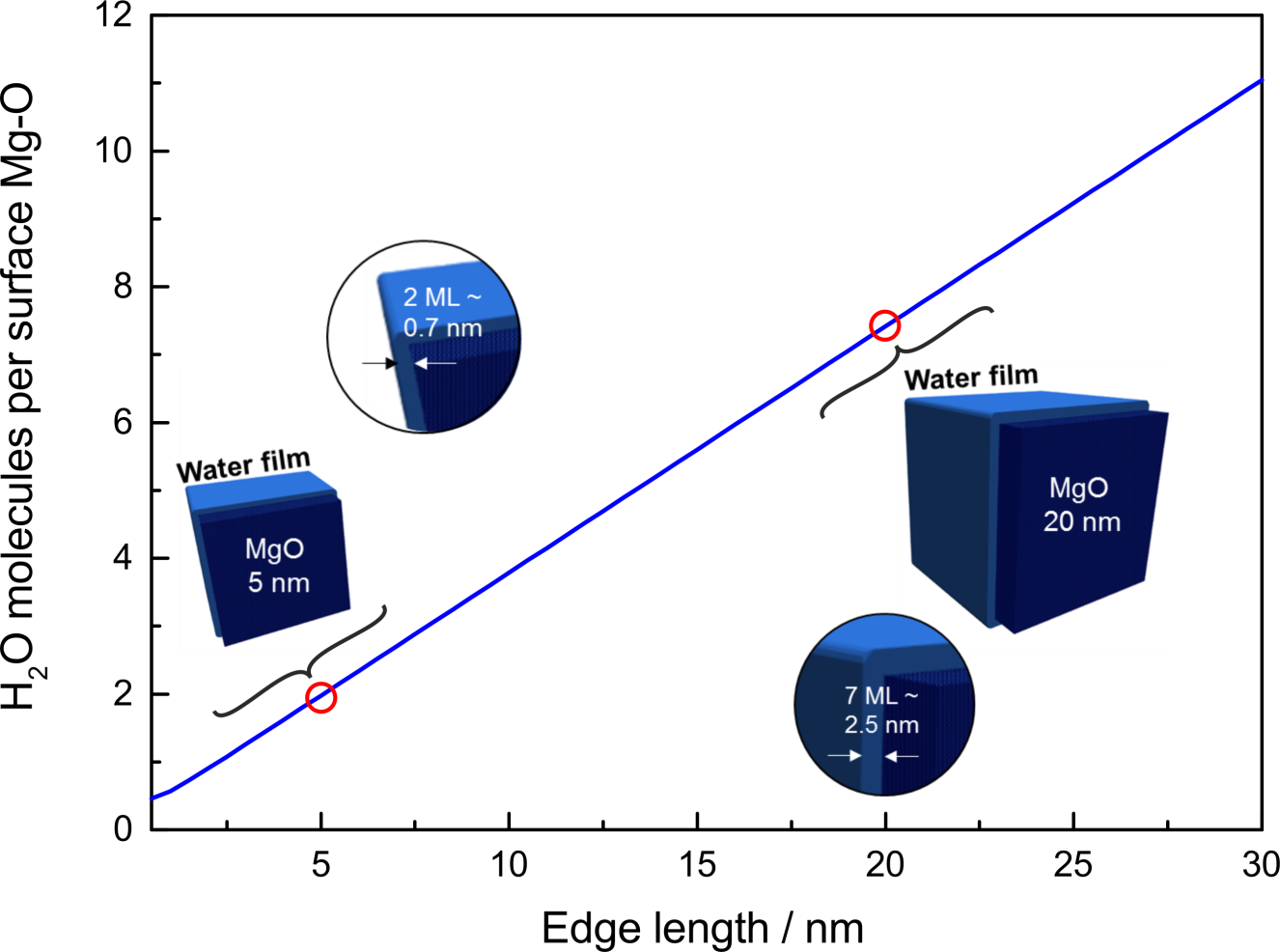
**

**Figure S3**: Estimated dependence of water film thickness on MgO nanocube size with a 16 wt-% loading of water on the particles.

Details about the calculations:

Lattice constant (MgO): a = 4.21·10^-10^ m

**MgO nanoparticles:**

Edge length: l_E_ = 5·10^-9^ m

Edge atoms: n_E_ = lE / (a · 0.5) = 5·10^-9^ m / (4.21·10^-10^ m · 0.5) = 24

Plane atoms: n_P_ = n_E_^2^ = 564

Surface atoms: n_S_ = (n_P_ - 4) · 6 – (n_E_ – 2) · 12 + 8 = 3108

Explanation: Six planes without corners - overlapping edges + corner atoms

Complete atoms: n_C_ = n_E_^3^ = 13402

Surface atom ratio: n_S_ / n_C_ = 23 %

**MgO sample:**

Mass (sample): m = 0.2 g

Molar mass (MgO): M(MgO) = 40.3 g/mol

H_2_O (ads.): w(H_2_O) = 16 wt.-%
